# Forceps Versus Embryulcia

**Published:** 1856-06

**Authors:** 


"*Forceps* versus *Embryulcia.*"

The article contained in the present number of the Journal from Dr. Freese, though very acceptable as a whole, yet contains one or two items which we are unwilling to let pass without a word of comment. So far as Dr. Freese inculcates the idea that the forceps should be used in all possible cases, in preference to the perforator, we fully coincide with him. From facts that have come under our own observation, we fear that this is not always the practice; but that embryotomy is sometimes resorted to, when a well-timed and skilful application of the forceps would have saved both mother and child. On the subject of compressing the child's head with the forceps, however, we should differ somewhat from our correspondent. We have in several instances where the labor had been protracted, and the child's head in consequence much elongated, applied the forceps and found the handles easily pressed together, and yet without compressing the head so much as to be in any degree injurious to the child.

On this subject we commend to all our readers the following paragraph from Dr. Meigs' Treatise on Obstetrics:—

"One of the most dangerous errors relative to the forceps that a Student could take up, would be the opinion that the forceps is, in its very design, a compressive instrument: it is not so; the forceps is not a pincers, it is an *extractor*—it is a real *tire-tete;* and I think it ought to be established as a *principle* in obstetrics, that where there is not space enough for the descent of the head without the forceps, *there cannot be produced a due proportion by merely squeezing the head down to the required dimensions by such an instrument.* Lest, however, I might by the above give a wrong impression of my views, it is needful that I should state, that a head, by long pressure of the pains, may be so moulded and reduced in diameter as to be squeezed through a pelvis smaller than the head was at the commencement of the travail: whenever, therefore, the pains cease, or are insufficient to produce it, the forceps, used as an extractor, may assist to that end; they should never squeeze it merely to compress and diminish its dimensions; they should always embrace it firmly enough to hold on and draw down, so that the passages may mould it as it descends."

"The above-mentioned results, procured by so distingushed a writer as Baudelocque, ought to suffice for removing any lingering disposition we might have to regard the forceps as a compressing instrument, and we should then be fully on our guard against the propensity to use it for such an object. But let it be considered that the head does not fill up the pelvis as a nail fills up the hole into which it is driven, but that it is always caught and arrested by two or perhaps four points on which it is impelled, and we shall see that if we do use it to squeeze and reduce the size of the head, we shall only reduce those diameters that are already small enough, and augment those that are already too large, for it cannot be adjusted on the points that are in such close contact as to constitute a real arrest. The most proper view to take of the instrument is, that it is a substitute for proper labor pain, supplying the want of expulsive force when wholly absent, or aiding it when its force is insufficient to effect the delivery of the woman."

